# Impact of different axial wall designs on the fracture strength and stress distribution of ceramic restorations in mandibular first molar

**DOI:** 10.1186/s12903-022-02577-5

**Published:** 2022-12-01

**Authors:** Bin Luo, Xiaolu Sun, Lin He, Lidan Zhao, Xinggang Liu, Qingsong Jiang

**Affiliations:** 1 Department of Prosthodontics, Beijing Stomatological Hospital, School of Stomatology, Capital Medical University, 100050 Beijing, China; 2 Shunyi Hospital, Beijing Traditional Chinese Medicine Hospital, 100010 Beijing, China; 3 Department of Prosthodontics, Affiliated Stomatology Hospital of Guangzhou Medical University, 510013 Guangzhou, China

**Keywords:** Ceramic restorations, Axial wall designs, Fracture strength, Stress distribution, Occlusal distal veneer

## Abstract

**Background:**

The purpose of this study was to investigate the fracture strength and stress distribution of four ceramic restorations.

**Methods:**

Forty human mandibular first molars were collected and randomized into four groups after establishing the distal defect: full crown group with 4 mm axial wall height (AWH) (FC4); short AWH crown group with 2 mm AWH (SC2); occlusal veneer group with 0 mm AWH (OV0); occlusal distal veneer group with only the distal surface prepared, and 4 mm AWH (OD4). The teeth were prepared according to the groups and the ceramic restorations were completed using celtra duo ceramic blocks. The ceramic thickness of the occlusal surface is about 1.5 mm and the edge is about 1 mm. The failure load values and fracture modes of each group were detected by mechanical test in vitro. According to the groups to establish three-dimensional finite element analysis (FEA) models, a 600 N loading force was applied vertically using a hemispherical indenter with a diameter of 6 mm. and compare the stress distribution under the condition of different restorations.

**Results:**

In vitro mechanical tests showed that the failure load values were SC2 (3232.80 ± 708.12 N) > OD4 (2886.90 ± 338.72 N) > VO0 (2133.20 ± 376.15 N) > FC4(1635.40 ± 413.05 N). The failure load values of the short AWH crown and occlusal distal veneer were significantly higher than that of occlusal veneer and full crown (P<0.05). The fracture modes of the full crown and occlusal veneer groups were mainly ceramic fractures and some were restorable tooth fractures. The short AWH crown and occlusal distal veneer groups presented with three fracture modes, the proportion of non-restorable tooth fracture was higher. The results of FEA show that under the spherical loading condition, the stress of ceramic was concentrated in the contact area of the loading head, the maximum von Mises stress values were FC4 (356.2 MPa) > VO0 (214.3 MPa) > OD4 (197.9 MPa) > SC2 (163.1 MPa). The stress of enamel was concentrated in the area where the remaining enamel was thinner, the maximum von Mises stress values was OD4 (246.2 MPa) ≈ FC4 (212.4 MPa) > VO0 (61.8 MPa) ≈ SC2 (45.81 MPa). The stress of dentin is concentrated in the root furcation and the upper third region of the root. However, stress concentration was observed at the tooth cervix in the full crown.

**Conclusion:**

Under certain conditions, the occlusal distal veneer shows better performance than the full crown.

## Background

Dental caries is a common oral disease. It is a chronic and progressive destructive disease occurring in the hard tissue of teeth under the influence of plaque and bacteria. According to the results of epidemiological survey, the prevalence of dental caries among Chinese adults was 62.7–76.5% in 2015 [1]. Molars are the most susceptible teeth to dental caries, and the proximal surface of molars is the common site of dental caries [2, 3]. Severe proximal caries can accumulate pulp and cause pulpitis or periapical inflammation. The affected tooth needs root canal therapy to eliminate the inflammation [4, 5]. Therefore, it is common to see teeth with proximal defects and have completed root canal therapy. For such teeth, in order to prevent secondary caries and protect the remaining dental tissue, it is often necessary to restore these defects with full crowns [6–8]. However, full crown restoration requires the removal of a large amount of normal tooth tissue[6, 9]. Research has shown that for the maxillary first molar, full crown preparation requires the removal of nearly 70% of the hard tissue of the crown [10]. Extensive cutting of the tooth tissue adversely affects long-term outcomes [11–13]. Therefore more minimally invasive restorative options need to be explored as an alternative to full crown restorations [14, 15].

With the development of all-ceramic materials and bonding techniques, a variety of minimally invasive restorative solutions have been proposed [16]. Minimally invasive ceramic restorations can be divided into two categories: embedded restorations represented by the endocrown [17–19], and extra-coronal restorations, including the short axial wall height (AWH) crown and occlusal veneer [15, 20–22]. The edge of the endocrown is butt joint design, which reduces the amount of tooth tissue that needs to be removed from the axial wall and presents higher fracture resistance [23, 24]. However, endocrown also has certain shortcomings. For example, the restoration is embedded into the pulp chamber to obtain retention, which will cause greater tensile stress on the dental tissue an increased risk of tissue fracture. In addition, the endocrown is difficult to remove, which makes it difficult to restore again [19]. The extra-coronal minimally invasive restorations can reduce the removal of the tooth tissue by reducing the coverage of the axial wall, and avoids embedding into the pulp chamber. A large number of studies have proved the feasibility of extra-coronal minimally invasive restorations. Studies by the Roberts group showed that the fracture strength of 2mmAWH crowns was comparable to that of full crowns in molars and premolars [25–27]. Studies by Zamzam and Ioanidis et al. proved that occlusal veneers can meet clinical requirements [28, 29]. In a recent study, a veneer covering the occlusal and buccal surface was used to restore severely worn teeth. The results of in vitro tests showed that the veneer covering the occlusal and buccal surface had higher fracture strength than the full crown [21]. In addition, tooth tissue removal can be reduced by reducing the thickness of the restorations. The study of Dal Piva AMO et al. showed that reducing the thickness of ceramic does not affect its mechanical behavior, reliability and translucency when selecting appropriate materials [30].

In clinical work, teeth that need to be restored are often accompanied by different degrees of axial wall defects. The design of restorations for teeth with axial wall defects is very important. The full crown can completely cover the defect but remove excessive tooth tissue. The extra-coronal minimally invasive restorations such as occlusal veneer cannot completely cover the defects, and the exposed fillings may have microleakage and secondary caries [31]. Therefore, there is no suitable restoration design for teeth with axial wall defects. Whether this occlusal veneer covering a single axial wall can be applied to teeth with proximal defects has not been studied.

The full crown is a classic crown restoration, short AWH crown and occlusal veneer are recommended minimally invasive ceramic restorations. To investigate the properties of occlusal distal veneer, we established axial wall defects in the distal surface of the teeth, and designed four types of restorations after root canal therapy. The performance of full crown, short AWH crown, occlusal veneer and occlusal distal veneer were compared. The fracture strength of different restorations was investigated by mechanical tests. At the same time, the three-dimensional finite element analysis (FEA) model is established to study the stress distribution. The null hypothesis is that there is no significant difference in stress distribution and in vitro failure load values between different restorations.

## Methods

### In vitro mechanical tests

**Fig. 1:**
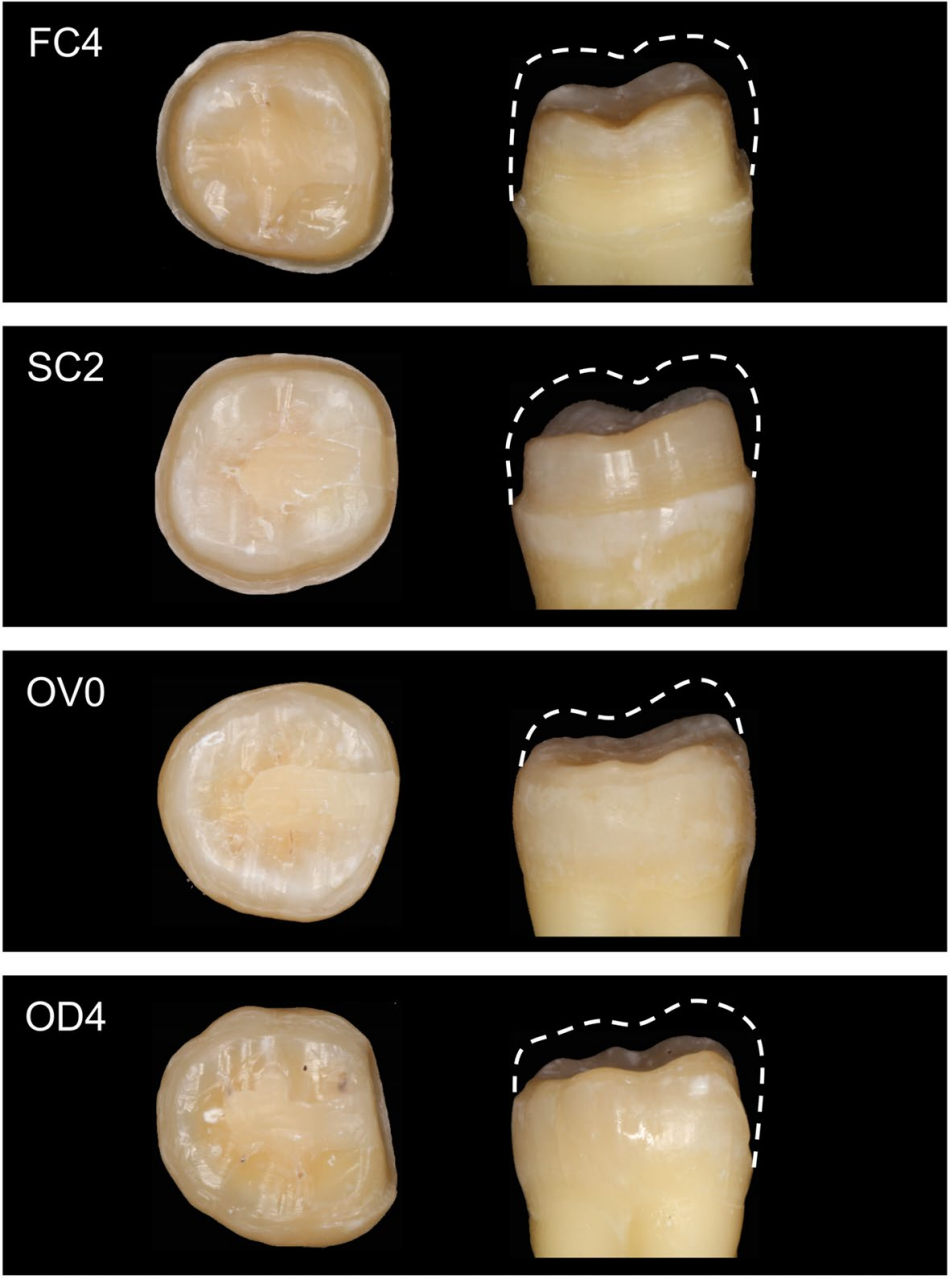
Schematic diagram of the preparation of each group of teeth. a full crown group with 4 mm AWH (FC4); short AWH crown group with 2 mm AWH (SC2); an occlusal veneer group with 0 mm AWH (OV0); occlusal distal veneer group with only the distal axial wall prepared, and 4 mm AWH (OD4)

Forty human mandibular first molars extracted due to periodontal disease and with similar crown dimensions were selected. After thorough cleaning, teeth were stored in 0.5% chloramine-T solution (Sigma-Aldrich, St. Louis, MO, USA) at 4℃. After establishing the distal defect and completing the root canal therapy, the teeth were randomly divided into 4 groups. Root canal therapy was performed according to a standardized procedure. First, the pulp chamber is opened with a high-speed bur, and then the root canal was unclogged with K files and the length of the root canal is measured. The Protaper system (Dentsply-Maillefer; Ballaigues, Switzerland) was used to complete the preparation of the root canal and the final file was F2. Root canal filling was done using gutta percha, and cone beam CT was taken to confirm the quality of Root canal therapy. The gingival wall of the distal defect was located 2 mm above the cemento-enamel junction (CEJ), with a width of 2 mm and a depth to pulp chamber, composite resin (Filtek Z250, 3 M, ESPE, USA) was used for filling. Group assignment was as follows: full crown group with 4 mm AWH (FC4); short AWH crown group with 2 mm AWH (SC2); occlusal veneer group with 0 mm AWH (OV0); occlusal distal veneer group with only the distal surface prepared, and 4 mm AWH (OD4) (Fig. 1). Tooth preparation was performed according to the design of different restorations, The occlusal surface was reduced to a thickness of 1.5 mm, and a 1 mm thick marginal chamfer was prepared 1 mm above the CEJ [32]. Tooth preparation was done using high-speed diamond burs (TR-21 ISO 197/016, TR-13 F ISO198/018, TR-26EF ISO199/016, EX-21EF ISO 237/019, MANI Inc., Amagasaki, Hyōgo Prefecture, Japan).

Cerec chairside digital equipment (CEREC AC Omnicam; Dentsply Sirona, Bensheim, Germany) was used to make an optical impression and generate a preparatory model. The restoration was generated by cerecSW4.4 (Dentsply Sirona, Bensheim, Germany) software, and the bonding gap was set to 100 μm. The restoration was designed digitally with a ceramic thickness of about 1.5 mm at the central fossa and 1 mm at the edge. The celtra Duo porcelain block (Dentsply Sirona, Bensheim, Germany) was used to complete the restoration. After cutting by the Wet-Milling Unit (Cerec MCXL, Dentsply Sirona, Bensheim, Germany), the restoration was sintered according to the manufacturer’s recommendations.

Restorations were bonded using Multilink N Kit (Ivoclar Vivadent, AG, Schaan, Lietschentein). All restorations were cleaned using ultrasound. The bonding surface was treated with hydrofluoric acid for 20s and then Monobond-S for 180s. Tooth preparations were etched with 37.5% phosphoric acid for 15 s, primer A and B were mixed 1:1 and then coated on the bonding surface of the teeth, and dried after 15s. Multilink N cement was coated on the bonding surface of the restorations, and then placed on the prepared tooth under 6 N load for 5 min. Marginal excess resin cement was removed after brief light curing with an Elipar™S10 curing light (3 M ESPE, St. Paul, Minnesota, USA). Then, each surface of the restorations was light cured for 20 s. Restorations stored in saline at 37° for 24 h, then subjected to 5000 thermal cycles in water between 5° and 55 °C on a thermal cycling simulator (Haake W15, SD Mechatronik, Feldkirchenen-Westerham, Germany) [33]. The residence time was 30 s and the transfer time between baths was 5 s [34]. The restorations were fixed in a 50 mm diameter resin disc for in vitro mechanical testing and did not simulate the periodontal ligament [35]. 6 mm steel balls were loaded at a loading speed of 0.5 mm/min [34], aluminums foil was placed between the loading ball and the restoration for buffering to avoid stress concentration, and the failure load values and failure mode were recorded [21, 36]. To ensure comparability between groups, all operations are performed by the same operator.

### Three-dimensional finite element analysis (FEA)

A human mandibular first molar extracted due to periodontal disease was selected after obtaining informed consent from the patient. The selected teeth were soaked in sodium hypochlorite liquid to disinfect and remove soft tissues, and the ultrasonic scaler was used to lightly remove dental calculus. Teeth were selected provided their structure was complete, without any cracks or caries. The anatomical shape of the occlusal surface was clear without any signs of serious wear. The shape of the tooth root was clearly and fully developed.

The tooth was embedded with resin and fixed in the scanning mold so that the long axis of the tooth was perpendicular to the scanning plane. A micro-computed tomography scan with a thickness of 0.019 mm was used to obtain the DICOM format data of the tooth. DICOM files were read to obtain three-dimensional images using Mimics16.0 software (Materialise, Leuven, Belgium). The enamel, dentin, periodontal ligament (PDL; thickness: 0.2 mm) and alveolar bone (Cortical bone thickness: 2 mm, Spongious bone thickness: 2 mm) models were established by adjusting the threshold and mesh repair step. The model after root canal therapy was established using SolidWorks2014 software (SolidWorks Corporation, Waltham, MA, USA). In addition, we designed distal defect, where the edge of the distal defect was 2 mm above the CEJ, and a resin filling module was established at the defect.

**Fig. 2:**
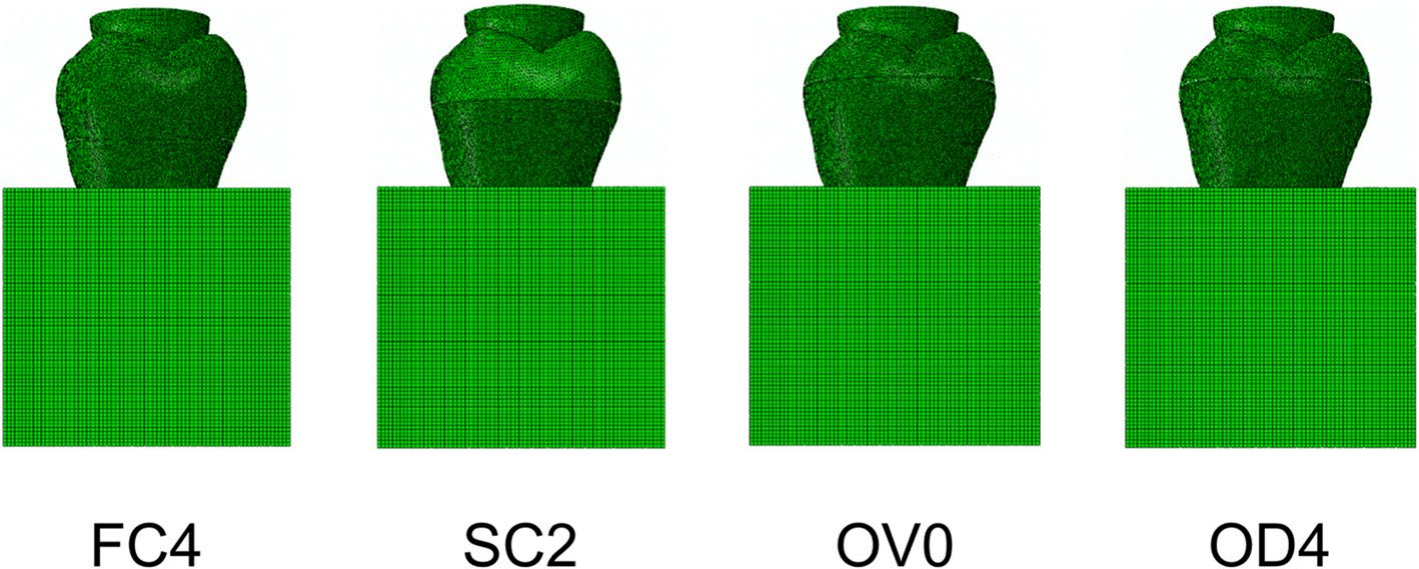
FEA model of each group. a full crown group with 4 mm AWH (FC4); short AWH crown group with 2 mm AWH (SC2); an occlusal veneer group with 0 mm AWH (OV0); occlusal distal veneer group with only the distal axial wall prepared, and 4 mm AWH (OD4)

After the tooth model was established, four types of ceramic restorations were established: FC4, full crown group with 4 mm AWH; SC2: short AWH crown group with 2 mm AWH; OV0: occlusal veneer group; OD4: occlusal distal veneer group (Fig. 2). The thickness of the ceramic restorations on the occlusal surface was of 1.5 mm, and 1 mm at the edge. A cement layer with a thickness of 100 μm was established between the restoration and tooth. Ceramic material with a Young’s modulus of 61 GPa and Poisson’s ratio of 0.3 µ. This experiment assumed that the materials used in the model were continuous, homogeneous and isotropic linear elastic. The solids of the FEA models are in fixed contact with each other. The mesh size is controlled at 0.2-0.4 mm, and the adjacent surface nodes are coupled by controlling the key parts. Mechanical properties of the materials used in FEA are presented in Table 1. The indenter was simulated as a hemispherical ball with a diameter of 6 mm to simulate the indenter used in the mechanical tests, and the teeth were vertically loaded using a 600 N loading force [37].

**Table 1:** Mechanical properties of the materials used in FEA

	Young’s modulus (GPa)	Poisson ratio(μ)	References
Enamel	84.1	0.33	[30]
Dentin	18.6	0.31	[21]
Cortical bone	13.7	0.3	[21]
Spongious bone	1.37	0.3	[21]
Multilink-N	8.3	0.35	[21]
Gutta percha	0.00069	0.45	[38]
Composite resin	15.8	0.24	[39]
Periodontal ligament	0.0689	0.45	[21]
Ceramic	61	0.3	[40]

#### Statistical analysis

The results of in vitro mechanical loading tests were compared among the groups using the analysis of variance, P < 0.05 were considered indicative of a statistically significant difference. In addition, we examined the failure modes of each group of restorations. FEA was used to compare the maximum von Mises stress and stress distribution among different restorations.

## Results

**Fig. 3:**
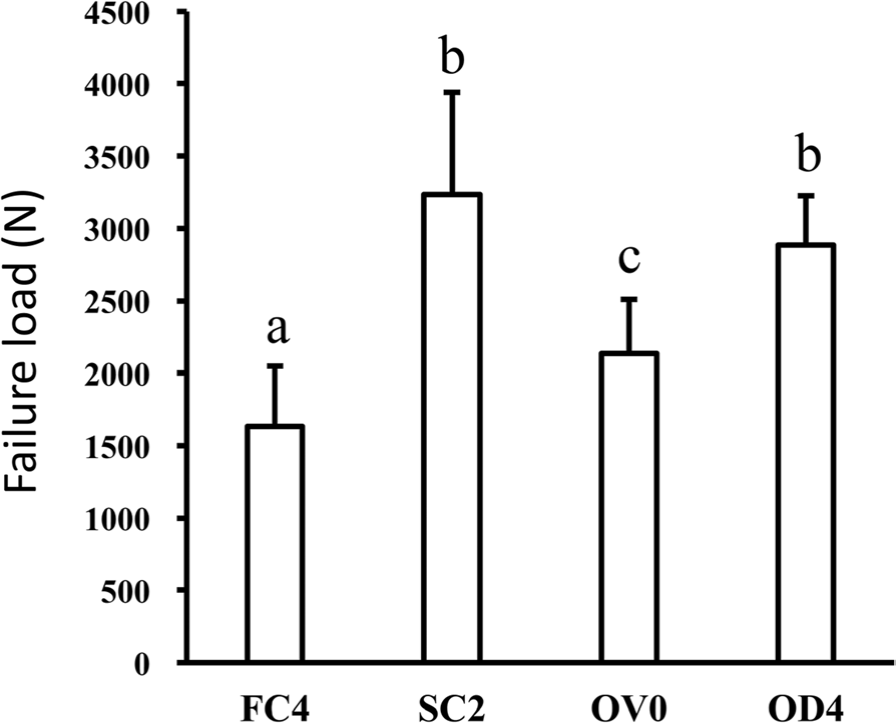
Mean failure load values of each group. The failure load values in each group were SC2 > OD4 > OV0 > FC4. The error bars represent on standard deviation (SD). The different letters indicate statistical differences between the groups (*P* < 0.05)

The results of in vitro mechanical tests showed that the failure load values were SC2 > OD4 > VO0 > FC4. The failure load values of the short AWH crown and occlusal distal veneer were significantly higher than that of occlusal veneer and full crown (*P*<0.05) (Fig. 3; Table 2).

**Table 2:** Mean failure load values (N) of each group

	Failure load values (N)
FC4	1635.40(413.05) ^a^
SC2	3232.80(708.12) ^b^
OV0	2133.20(376.15) ^c^
OD4	2886.90(338.72) ^b^

*Note*: The different letters indicate statistical differences between the groups (*P*< 0.05)

**Fig. 4:**
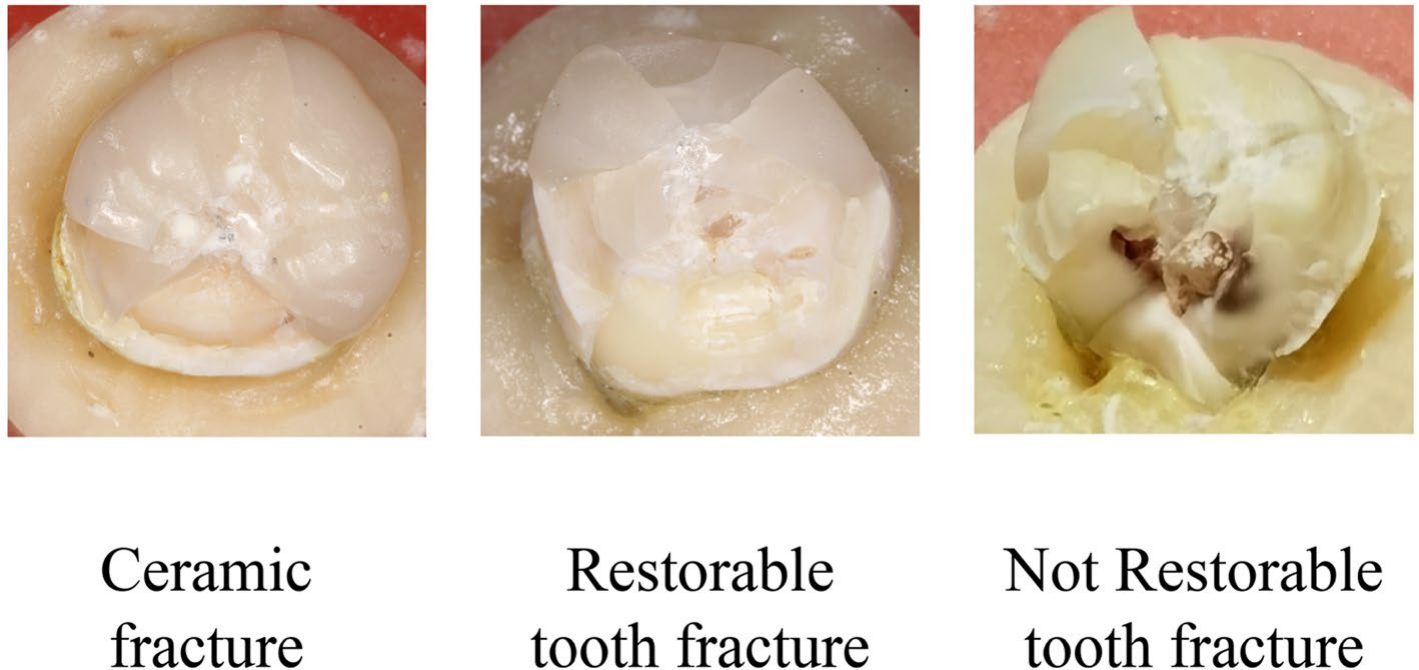
Failure Mode Analysis. The fracture mode was divided into ceramic fracture, restorable tooth fracture and non-restorable tooth fracture

Depending on whether the fracture exceeded the CEJ, the fracture modes were divided into ceramic fracture, restorable tooth fracture, and non-restorable tooth fracture [36] (Fig. 4). The fracture modes of the full crown and occlusal veneer groups were mainly ceramic fractures and some were restorable tooth fractures. The short AWH crown and occlusal distal veneer groups presented with three fracture modes, the proportion of non-restorable tooth fracture was higher (Table 3).

**Table 3:** Failure mode analysis of each group

	Failure mode		
	Ceramic fracture	Restorable tooth fracture	Not Restorable tooth fracture
FC4	9	1	0
SC2	2	3	5
OV0	6	4	0
OD4	3	2	5

**Fig. 5:**
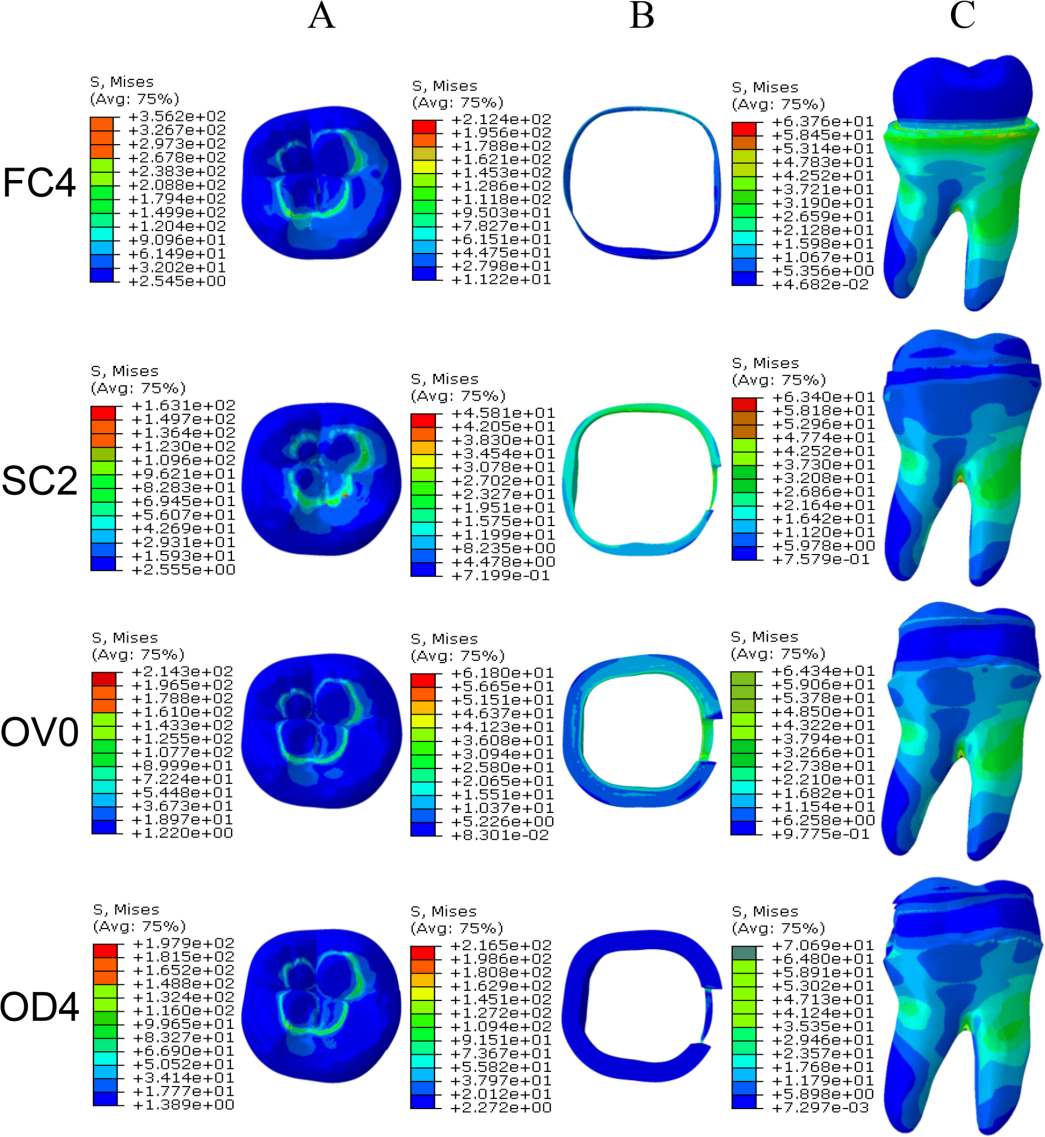
Cloud atlas of the maximum von Mises stress (MPa) on the (**A**) ceramic, (**B**) enamel and (**C**) dentin of each group. Under the spherical loading condition, the stress in each group of ceramic was concentrated in the contact area of the loading head, enamel stress was concentrated in the area where the remaining enamel was thinner, dentin stress was concentrated in the root furcation and the upper third region of the root. However, stress concentration was observed at the tooth cervix in the full crown

The results of FEA show that under the spherical loading condition, the stress of ceramic was concentrated in the contact area of the loading head (Fig. 5 A), among-group relationships of the maximum von Mises stress values were FC4 > VO0 > OD4 > SC2 (Table 4). The stress of enamel was concentrated in the area where the remaining enamel was thinner, and the relationship between the groups of the maximum von Mises stress values was OD4 ≈ FC4 > VO0 ≈ SC2 (Fig. 5B; Table 4). The stress of dentin is mainly concentrated in the root furcation and the upper third region of the root. However, stress concentration was observed at the tooth cervix in the full crown, and the maximum von Mises stress was about FC4 ≈ SC2 ≈ VO0 ≈ OD4 (Fig. 5 C and Table 4).

**Table 4:** Maximum von Mises stress (MPa) of each group

		maximum von Mises stress (MPa)		
		Enamel	Dentin	Ceramic
CeramicY=61.0GPaP=0.3	FC4	212.4	63.76	356.2
	SC2	45.81	63.4	163.1
	OV0	61.8	67.8	214.3
	OD4	246.2	70.69	197.9

## Discussion

With the development of bonding technology and ceramic materials, minimally invasive restoration has become a focus of attention and research in the field of tooth restoration [41, 42]. In this study, we established axial wall defects in the distal surface of the tooth, and compared the fracture strength and stress distribution of four different restorations. Our results showed that the failure load values of the short AWH crown and occlusal distal veneer were significantly higher than that of the occlusal veneer, and the occlusal veneer were significantly higher than that of the full crown.

The concept of minimally invasive restoration is to pursue the preservation of more normal tooth tissue. A large number of studies have confirmed that the preservation of more normal tooth tissue is beneficial to the mechanical properties of teeth [23, 43, 44]. The short AWH crown, occlusal veneer and occlusal distal veneer in this study are minimally invasive restorations. Compared with the full crown, the tooth tissue was removed less, and the preserved tooth tissue improved the fracture strength, which was consistent with the results of previous studies [45]. Full crowns remove too much tooth tissue during tooth preparation, especially the tooth cervix [9]. The results of FEA showed that there was stress concentration in the tooth cervix after full crown restoration, which further explained the reason for the lowest failure load values of full crown. The studies of Roberts group showed that the fracture strength of full crown and short AWH crown was similar, which was not consistent with this test. The possible reasons for the difference include whether the molars performed root canal therapy, whether the distal defects were present and ceramic material [25, 26]. The failure load values between the short AWH crown, occlusal veneer and occlusal distal veneer were inconsistent in previous studies. Jenista et al. showed that the failure load values of the 2 mm short AWH crown was greater than that of occlusal veneer in molars [46]. Taha et al. showed that endocrown with 1 mm axial wall exhibited higher failure load values [34]. Huang et al. showed that the failure load values of premolar occlusal veneer was greater than that of veneer covering the occlusal and buccal surface [21]. Our research results show that the fracture resistance of the short AWH crown and the occlusal distal veneer is higher than that of the occlusal veneer. We believe that a small amount of axial wall design provides a moderately increased bonding area and support for the restorations. However, excessive removal of tooth tissue, especially in the tooth cervix, will damage the fracture strength of the restorations and tooth.

The fracture mode analysis results show that the short AWH crown and occlusal distal veneer groups have more non-restorable tooth fracture, which is related to higher failure load values. The highest failure load value of the full crown group was 2165 N, while the failure load values of all samples in the short AWH crown group and occlusal distal veneer groups were higher than 2165 N. Therefore, the increase in the proportion of non-restorable tooth fracture may be due to excessive loading. The occlusal load of the human molar has been estimated in the range of 100–200 N, although it can reach 965 N in accidental bites or trauma. Therefore, a failure load greater than 1000 N is required for good clinical performance [26]. In this study, the failure load of each group was greater than 1000 N, satisfying this requirement.

The literature review of in vitro mechanical testing of ceramic restorations showed that the failure load of the full crown was 1034.8-2939 N, the short AWH crown was 930-1034 N, and the occlusal veneer was 1191-3584 N [15, 25, 26, 47]. The failure loads of full crowns and occlusal veneers in this test are consistent with previous studies. There are few literatures about short AWH crown. The failure load of short AWH crown in this test is higher than that in previous studies. Possible reasons include differences in materials, thickness, loading conditions and aging conditions, etc. The occlusal distal veneer has not been reported in the literature. In this study, the occlusal distal veneer showed good performance.

FEA is an effective method to analyze the stress distribution of restorations. Commonly used parameters include maximum principal stress and maximum von Mises stress. The maximum principal stress reflects the maximum force in a single direction, and the maximum von Mises stress is the synthesis of various stresses in the model to reflect the overall stress at the stressed area [48]. Many studies have suggested that FEA can be used to observe the stress distribution of teeth to assist the analysis of in vitro mechanical test results, and the results of the two methods have a good consistency [49, 50]. In this study, the results of in vitro tests showed that the failure load values of the short AWH crown and occlusal distal veneer were similar. The results of FEA showed that the stress distribution and maximum von Mises stress of ceramic and dentin in the short AWH crown and the occlusal distal veneer were similar, but the maximum von Mises stress of enamel in the occlusal distal veneer was significantly higher than that in the short AWH crown and similar to that in the full crown. The FEA model designed in this study was a tooth with a distal defect. In order to cover the distal filling, the distal surface removal and restoration coverage area of the occlusal distal veneer was consistent with full crown. Therefore, the maximum von Mises stress in the remaining enamel was similar to that in the full crown, but the occlusal distal veneer did not show the stress concentration in the tooth cervix because the integrity of the other three walls was preserved. The studies of Beata Dejak and Lin also support that the full crown is more prone to stress concentration in the cervix [51, 52].

The occlusal distal veneer has many advantages, the edge of the occlusal distal veneer is short and close to the crown, supporting daily cleaning and helping reduce the risk of secondary caries and periodontal inflammation. In addition, the edge of the occlusal distal veneer that is close to the Occlusal surface helps obtain clear scanning data, easier bonding and moisture separation [53]. Compared with the margin elevation technology, the occlusal distal veneer adopts indirect repair to obtain better edge sealing and reduce the possible impact of resin aging [54]. All in all, this research found that the occlusal distal veneer is in line with the concept of minimally invasive restoration and shows better mechanical properties than full crowns. Under certain circumstances, it can meet the needs of clinical restorations and can be used as a reference plan for clinical restoration design. Of course, this study still has some limitations. We only examined the performance of different restorations under vertical loading. However, in the actual situation, the masticatory load is in different directions, and the performance of the restorations under different loading conditions still needs to be explored. In addition, clinical trials are more convincing to test the performance of the restorations, and the reliability and potential problems of the occlusal distal veneer still need to be demonstrated through clinical trials.

## Conclusion

Under certain conditions, the occlusal distal veneer shows better performance than the full crown, and can meet the strength requirements of clinical restorations, which can be used as a reference for the design of clinical restorations.

## Data Availability

All data generated or analysed during this study are included in this published article.

## Abbreviations

AWH: Axial wall height
FEA: Three-dimensional finite element analysis
CEJ: Cemento-enamel junction
PDL: Periodontal ligament
